# Sex-specific associations between daytime sleepiness, chronic diseases and mortality in obstructive sleep apnea

**DOI:** 10.3389/fnins.2023.1210206

**Published:** 2023-06-23

**Authors:** Naima Covassin, Dongmei Lu, Erik K. St. Louis, Anwar A. Chahal, Phillip J. Schulte, Meghna P. Mansukhani, Jiang Xie, Melissa C. Lipford, Nanfang Li, Kannan Ramar, Sean M. Caples, Peter C. Gay, Eric J. Olson, Michael H. Silber, Jingen Li, Virend K. Somers

**Affiliations:** ^1^Department of Cardiovascular Medicine, Mayo Clinic, Rochester, MN, United States; ^2^Department of Respiratory and Critical Care Medicine, People's Hospital of Xinjiang Uygur Autonomous Region, Urumqi, China; ^3^Department of Medicine, Mayo Clinic, Rochester, MN, United States; ^4^Department of Neurology, Mayo Clinic, Rochester, MN, United States; ^5^Center for Sleep Medicine, Division of Pulmonary and Critical Care Medicine, Mayo Clinic, Rochester, MN, United States; ^6^Clinical Trials and Biostatistics, Department of Quantitative Health Sciences, Mayo Clinic, Rochester, MN, United States; ^7^Department of Family Medicine, Mayo Clinic, Rochester, MN, United States; ^8^Department of Respiratory and Critical Medicine of Beijing An Zhen Hospital, Capital Medical University, Beijing, China; ^9^Center of Hypertension of the People's Hospital of Xinjiang Uygur Autonomous Region, The Center of Diagnosis, Treatment and Research of Hypertension in Xinjiang Hypertension Institute of Xinjiang, Urumqi, China; ^10^Department of Cardiovascular Medicine, Dongzhimen Hospital, Beijing University of Chinese Medicine, Beijing, China

**Keywords:** sleepiness, obstructive sleep apnea, mortality, sex differences, chronic disease

## Abstract

**Objective:**

Excessive daytime sleepiness (EDS) is common in obstructive sleep apnea (OSA) and has been linked to adverse outcomes, albeit inconsistently. Furthermore, whether the prognostic impact of EDS differs as a function of sex is unclear. We aimed to assess the associations between EDS and chronic diseases and mortality in men and women with OSA.

**Methods:**

Newly-diagnosed adult OSA patients who underwent sleep evaluation at Mayo Clinic between November 2009 and April 2017 and completed the Epworth Sleepiness Scale (ESS) for assessment of perceived sleepiness (*N* = 14,823) were included. Multivariable-adjusted regression models were used to investigate the relationships between sleepiness, with ESS modeled as a binary (ESS > 10) and as a continuous variable, and chronic diseases and all-cause mortality.

**Results:**

In cross-sectional analysis, ESS > 10 was independently associated with lower risk of hypertension in male OSA patients (odds ratio [OR], 95% confidence interval [CI]: 0.76, 0.69–0.83) and with higher risk of diabetes mellitus in both OSA men (OR, 1.17, 95% CI 1.05–1.31) and women (OR 1.26, 95% CI 1.10–1.45). Sex-specific curvilinear relations between ESS score and depression and cancer were noted. After a median 6.2 (4.5–8.1) years of follow-up, the hazard ratio for all-cause death in OSA women with ESS > 10 compared to those with ESS ≤ 10 was 1.24 (95% CI 1.05–1.47), after adjusting for demographics, sleep characteristics and comorbidities at baseline. In men, sleepiness was not associated with mortality.

**Conclusion:**

The implications of EDS for morbidity and mortality risk in OSA are sex-dependent, with hypersomnolence being independently associated with greater vulnerability to premature death only in female patients. Efforts to mitigate mortality risk and restore daytime vigilance in women with OSA should be prioritized.

## 1. Introduction

Excessive daytime sleepiness (EDS) is a debilitating complaint reported by 9–20% of the general population (Empana et al., 2009; Boden-Albala et al., 2012; Ohayon, 2012). Hypersomnolence has significant consequences for daily functioning, compromising work productivity and quality of life and increasing risk of motor vehicle and occupational accidents (Dean et al., 2010; Ohayon, 2012; Bioulac et al., 2017). Moreover, accumulating evidence suggests that EDS may adversely impact health, with studies linking hypersomnolence to diabetes (Bixler et al., 2005; Empana et al., 2009; Blachier et al., 2012; Vashum et al., 2015), coronary heart disease (Newman et al., 2000; Boden-Albala et al., 2012), stroke (Blachier et al., 2012; Boden-Albala et al., 2012; Vashum et al., 2015), depression (Bixler et al., 2005; Empana et al., 2009; Ohayon, 2012), and cancer (Ohayon, 2012; Jaumally et al., 2021). EDS has also been found to prognosticate greater risk of cardiovascular and total mortality in population-based studies (Newman et al., 2000; Empana et al., 2009; Boden-Albala et al., 2012; Li et al., 2021).

A pathological tendency to fall asleep is traditionally regarded as the cardinal symptom of obstructive sleep apnea (OSA), a common disorder affecting 34% of men and 17% of women (Peppard et al., 2013). OSA manifests with recurrent episodes of partial or complete collapse of the upper airway during sleep, leading to increased respiratory effort, hypoxemia, sympathoexcitation, and sleep fragmentation. In patients with OSA, EDS is thought to ensue primarily from sleep disruption consequent to abnormal respiratory events, although inflammation, comorbidities or even genetics may contribute (Garbarino et al., 2018). Akin to evidence from the general population, associations between hypersomnolence and excess disease risk have been reported in OSA, including hypertension, diabetes, cardiovascular disease (CVD), and depression (Koutsourelakis et al., 2008; Ronksley et al., 2009). However, other studies did not corroborate such findings (Kapur et al., 2005; Tam et al., 2019). Similarly, data on the relation between EDS and survival in OSA are limited and discordant (Young et al., 2008; Xie et al., 2018; Mazzotti et al., 2019; Trzepizur et al., 2022). Although such discrepancy may be due to several aspects, including the various definitions of EDS and assessment modalities, sex differences may play a role.

OSA exhibits a well-known sex patterning, with women making up a smaller proportion of the OSA population especially at younger ages (Peppard et al., 2013). Pathophysiology and clinical presentation of OSA vary by sex, with women being more likely to report symptoms such as fatigue, depression, and insomnia, and to exhibit lower frequency and duration of apneic events and less intense snoring (Bonsignore et al., 2019). Evidence on whether EDS associated with OSA differs between the sexes is conflicting (Kapur et al., 2005; Koutsourelakis et al., 2008; Huang et al., 2018; Nigro et al., 2018) and, importantly, the interplay between sleepiness and sex in relation to health outcomes has not been systematically studied in OSA. To this end, few investigations have found EDS to be more closely associated with poor health in men with OSA (Basta et al., 2008; Aurora and Punjabi, 2019), while others have reported stronger effects in women with OSA (Huang et al., 2018; Kendzerska et al., 2020). The implications for long-term outcomes are also largely unknown.

We therefore examined sex-specific associations between EDS and prevalence of chronic diseases in a large sample of OSA patients. In a longitudinal analysis, we investigated whether EDS predicts mortality in this group of men and women with OSA (primary endpoint).

## 2. Materials and methods

### 2.1. Study population

This retrospective, single-center study examined a consecutive sample of adults with suspected sleep disorders who underwent a diagnostic polysomnography (PSG) at the Mayo Clinic Center for Sleep Medicine between November 17th, 2009 and April 15th, 2017. From the initial sample (*N* = 30,903), we excluded patients aged <18 years old (*n* = 4,473), patients without research authorization (*n* = 1,485), those who underwent non-diagnostic (*n* = 1,240) or follow-up PSGs (*n* = 531), and those for whom >3 months elapsed between sleep consultation and PSG (*n* = 2,174) (Lipford et al., 2019). We then excluded those who did not complete the Epworth Sleepiness Scale (ESS) for somnolence assessment (*n* = 1,299), those without OSA (i.e., apnea-hypopnea index [AHI] < 5 events/h; or with central sleep apnea, namely central apnea index ≥5 and > 50% of AHI; *n* = 4,304) (American Academy of Sleep Medicine, 2014), those with duration of recording <2 h (*n* = 513), total sleep time < 1 h (*n* = 46) (Nigro et al., 2018; Earl et al., 2019), and those pregnant (*n* = 15) at the time of PSG. The analytical sample for the cross-sectional examination consisted of 14,823 OSA patients with ESS data. Patients without follow-up information (*n* = 78) were further excluded for the longitudinal analysis, yielding a total of 14,745 patients for mortality assessment (Supplementary Figure 1). The study was approved by the Mayo Clinic Institutional Review Board and research authorization was verified for all participants (Minnesota Statute 144.295).

### 2.2. Sleep assessment

In-laboratory PSGs were performed and analyzed using Nicvue (Nicolet, Inc., Middleton, WI). PSG montage included electroencephalography, right and left electrooculography, submental and limb electromyography, electrocardiography, oronasal thermistor, nasal flow pressure sensor, thoracic and abdominal inductance plethysmography, position sensor, pulse oximetry and sound recording. Sleep recordings were scored according to the American Academy of Sleep Medicine criteria (Iber et al., 2007) by a registered polysomnographic technologist and reviewed by a board-certified sleep medicine specialist. Apneas were defined as ≥90% reductions in airflow lasting for ≥10 s. Hypopneas were scored when ≥30% decreases in airflow occurred for ≥10 s and were accompanied by ≥4% oxyhemoglobin desaturation. AHI was calculated as the sum of apneas and hypopneas normalized by sleep time. OSA severity was further classified as mild (AHI 5–14.9 events/h), moderate (AHI 15–29.9 events/h), and severe (AHI ≥ 30 events/h). Additional variables derived from PSG included total recording time, total sleep time, sleep efficiency, arousal index, periodic limb movement index (PLMI), mean oxyhemoglobin saturation (SpO_2_), minimum SpO_2_, and percentage of total sleep time spent with SpO_2_ < 90% (T90, %). The majority of PSGs (94.7%) were conducted in a split-night fashion.

Daytime somnolence was evaluated by ESS (Johns, 1991), a self-report instrument assessing a subject’s likelihood of falling asleep in 8 daily situations. Each item is rated on a scale from 0 (no chances of dozing) to 3 (high chances of dozing). Higher score indicates higher levels of habitual daytime sleepiness, and a score > 10 is suggestive of EDS (Johns, 1991).

### 2.3. Demographic, clinical characteristics and follow-up

Demographic and clinical variables were abstracted from the electronic medical records. Race was coded as White or non-White. Height and weight for body mass index (BMI) calculations were obtained prior to PSG. Positive smoking history was defined as current or previous smoking. International Classification of Diseases codes were used to identify comorbid diagnosis of hypertension, diabetes mellitus, CVD, chronic obstructive pulmonary disease (COPD), chronic kidney disease, liver disease, cancer, depression and insomnia (Supplementary Table 1), with positive cases defined as at least two codes noted at different dates. Hypnotics usage was similarly obtained.

For each patient, survival status was monitored from the PSG date to the date of death or April 29^th^, 2021, whichever occurred first, and ascertained using the Accurint system (all States death records certification system) and the Mayo Clinic electronic medical records. Because quantitative compliance data with therapy during the follow-up could not be obtained, we defined positive airway pressure (PAP) treatment acceptance based on evidence of prescription after OSA diagnosis and at least one subsequent note confirming usage, as previously described (Gami et al., 2013; Kendzerska et al., 2020).

### 2.4. Statistical analysis

Continuous variables are described as medians and interquartile range (IQR), and categorical variables are reported as frequency and percentage. Patient characteristics were compared between those with (ESS > 10) and without (ESS ≤ 10) EDS using Mann–Whitney U test and Pearson Chi-square test where appropriate, separately in men and women.

Multivariable-adjusted sex-specific odds ratio (OR) and 95% confidence interval (CI) of the relation between ESS > 10 and chronic diseases were obtained using logistic regression analysis. Kaplan–Meier curves and log-rank tests described univariate survival rates in sleepy vs. non-sleepy men and women, while Cox proportional hazard regression models were constructed to assess the independent association between ESS > 10 and all-cause mortality. Residuals were inspected to verify proportional hazards assumptions, and results are reported as hazard ratio (HR) with 95% CI. For both cross-sectional and longitudinal analyses, adjusted estimates with ESS score modeled as a continuous variable were also obtained. Multivariable restricted cubic spline regression was used to explore nonlinear functional relationships, with knots placed at 10th, 50th and 90th percentile of ESS score. To assess effect modifiers of the association between EDS and outcomes in both sexes, interaction effects were tested and stratified analyses were conducted across age (<65, ≥65 years), BMI (<30, ≥30 kg/m^2^) and OSA severity (AHI 5–14.9, 15–29.9, ≥30 events/h) categories. No correction for multiple comparisons was applied.

We tested the robustness of our findings by conducting several sensitivity analyses. To test for the potential confounding effects of unmeasured factors on mortality, we excluded deaths within 6 and 12 months from PSG. We also excluded patients with CVD and cancer at baseline. In multivariable analysis, we further corrected for PAP acceptance and restricted the sample to split-night studies. Lastly, we compared characteristics between patients with and without available ESS to assess potential for selection bias.

Statistical significance was set at *p* < 0.05. Analyses were performed using SPSS 25 (IBM Inc.) and R (version 3.4.2).

## 3. Results

### 3.1. Baseline characteristics

The median age of the sample was 61 years (IQR 51, 70 years) and 38.7% were women (Supplementary Table 2). Men had lower BMI and were more likely to report a history of smoking than women. Arousal index, PLMI, AHI and T90 were higher while sleep efficiency was lower in men than in women. Men were more likely to have CVD, chronic kidney disease, and cancer than women. Conversely, depression and insomnia were more frequent among women.

ESS > 10 (EDS) was reported by 40.2% of men and 39.4% of women. Both men and women with EDS were younger, had higher BMI and were more likely to have severe OSA compared to their counterparts without EDS (Tables 1, 2). Hypertension, CVD and insomnia were less frequent while diabetes mellitus was more frequent among men and women with EDS than in those without. Depression was more common in women with EDS than in women without it.

**Table 1:** Characteristics of OSA men with and without EDS.

Characteristic	Total (*n* = 9,081)	ESS > 10 (*n* = 3,647)	ESS ≤ 10 (*n* = 5,434)	*p* value
Age, years	60 (50, 70)	59 (49, 70)	61 (51, 70)	<0.001
White, *n* (%)	8,264 (92.8)	3,284 (91.8)	4,980 (93.5)	0.002
BMI, kg/m^2^	31.9 (28.5, 35.9)	32.1 (28.7, 36.0)	31.7 (28.4, 35.9)	0.005
Smoking history, *n* (%)	3,031 (33.6)	1,220 (33.7)	1,811 (33.6)	0.922
Sleep measures
ESS score	9 (5, 13)	14 (12, 17)	6 (4, 8)	< 0.001
Split-night, n (%)	8,642 (95.2)	3,492 (95.7)	5,150 (94.8)	0.033
Total recording time, min	222 (182, 272)	216 (178, 266)	226 (185, 274)	< 0.001
Total sleep time, min	153 (129, 183)	153 (129, 183)	154 (129, 183)a	0.768
Sleep efficiency, %	74.9 (61.8, 84.6)	76.9 (63.4, 86.1)	73.8 (60.9, 83.6)	< 0.001
Arousal index, events/h	39.2 (26.1, 59.3)	40.5 (26.6, 61.9)	38.4 (25.9, 57.6)	0.002
PLMI, events/h	18.3 (0.9, 60.2)	17.0 (0.4, 59.4)	19.2 (1.1, 60.7)	0.103
AHI, events/h	20 (10, 40)	21 (10, 44)	19 (9, 37)	< 0.001
RDI, events/h	30 (17, 53)	32 (18, 57)	29 (17, 50)	< 0.001
Mean SpO_2_, %	93 (91, 94)	93 (91, 94)	93 (91, 94)	0.116
Minimum SpO_2_, %	83 (78, 86)	83 (77, 86)	83 (78, 86)	0.007
T90_,_ %	5.1 (1.3, 17.6)	5.2 (1.3, 18.7)	5.0 (1.2, 17.0)	0.079
OSA severity				< 0.001
AHI 5–14.9, *n* (%)	3,500 (38.5)	1,320 (36.2)	2,180 (40.1)	
AHI 15–29.9, *n* (%)	2,353 (25.9)	922 (25.3)	1,431 (26.3)	
AHI ≥ 30, *n* (%)	3,228 (35.5)	1,405 (38.5)	1,823 (33.5)	
Comorbidities
Hypertension, *n* (%)	4,410 (48.6)	1,616 (44.3)	2,794 (51.4)	< 0.001
Diabetes mellitus, *n* (%)	1,868 (20.6)	799 (21.9)	1,069 (19.7)	0.010
CVD, *n* (%)	3,001 (33.0)	1,140 (31.3)	1,861 (34.2)	0.003
COPD, *n* (%)	639 (7.0)	249 (6.8)	390 (7.2)	0.523
Chronic kidney disease, *n* (%)	733 (8.1)	297 (8.1)	436 (8.0)	0.837
Liver disease, *n* (%)	644 (7.1)	273 (7.5)	371 (6.8)	0.231
Cancer, *n* (%)	1,097 (12.1)	431 (11.8)	666 (12.3)	0.530
Depression, *n* (%)	209 (2.3)	90 (2.5)	119 (2.2)	0.387
Insomnia, *n* (%)	1,008 (11.1)	372 (10.2)	636 (11.7)	0.025
Hypnotics usage, *n* (%)	790 (8.7)	256 (7.0)	534 (9.8)	< 0.001
Follow up, years	6.2 (4.4, 8.1)	6.2 (4.4, 8.0)	6.2 (4.5, 8.1)	0.226
PAP acceptance, %	4,909 (54.4)	1,966 (54.2)	2,943 (54.6)	0.711

Data from continuous variables are reported as median (IQR) values and count (%).

AHI, apnea-hypopnea index; BMI, body mass index; COPD, chronic obstructive pulmonary disease; CVD, cardiovascular disease; EDS, excessive daytime sleepiness; ESS, Epworth Sleepiness Scale; IQR, interquartile range; OSA, obstructive sleep apnea; PAP, positive airway pressure; PLMI, periodic limb movement index; RDI, respiratory disturbance index; SpO_2_, oxyhemoglobin saturation; T90, total sleep time (in percent) spent with SpO_2_ below 90%.

Numbers may not sum to totals because of missing data.

*p*-values from Mann–Whitney *U* test or Pearson Chi square.

**Table 2:** Characteristics of OSA women with and without EDS.

Characteristic	Total (*n* = 5,742)	ESS > 10 (*n* = 2,265)	ESS ≤ 10 (*n* = 3,477)	*p* value
Age, years	61 (51, 70)	58 (49, 68)	63 (53, 71)	< 0.001
White, *n* (%)	5,240 (92.4)	2,063 (92.2)	3,177 (92.5)	0.655
BMI, kg/m^2^	34.2 (29.3, 40.2)	34.8 (29.8, 40.6)	33.7 (28.9, 39.8)	< 0.001
Smoking history, *n* (%)	1,558 (27.3)	655 (29.1)	903 (26.1)	0.013
Sleep measures				
ESS score	9 (5, 13)	14 (12, 17)	6 (3, 8)	< 0.001
Split-night, *n* (%)	5,397 (94.0)	2,144 (94.7)	3,253 (93.6)	0.086
Total recording time, min	240 (196, 292)	236 (192, 287)	242 (200, 296)	< 0.001
Total sleep time, min	168 (139, 209)	170 (141, 212)	166 (138, 207)	0.028
Sleep efficiency, %	76.5 (63.7, 85.7)	78.5 (66.3, 87.2)	75.0 (62.1, 84.7)	< 0.001
Arousal index, events/h	31.3 (20.1, 47.9)	31.5 (20.0, 48.8)	31.1 (20.2, 47.4)	0.366
PLMI, events/h	11.1 (0.0, 42.5)	9.1 (0.0, 39.0)	12.5 (0.0, 44.1)	0.003
AHI, events/h	13 (7, 26)	13 (7, 29)	13 (7, 25)	0.050
RDI, events/h	22 (13, 39)	23 (13, 41)	21 (13, 38)	0.052
Mean SpO_2_, %	93 (91, 94)	93 (91, 94)	93 (91, 94)	0.570
Minimum SpO_2_, %	83 (78, 86)	83 (78, 86)	83 (78, 86)	0.870
T90, %	4.0 (1.0, 17.5)	4.0 (0.9, 19.1)	4.1 (1.1, 16.8)	0.433
OSA severity				0.007
AHI 5–14.9, *n* (%)	3,124 (54.4)	1,201 (53.0)	1,923 (55.3)	
AHI 15–29.9, *n* (%)	1,361 (23.7)	520 (23.0)	841 (24.2)	
AHI ≥ 30, n (%)	1,257 (21.9)	544 (24.0)	713 (20.5)	
Comorbidities				
Hypertension, *n* (%)	2,747 (47.8)	1,028 (45.4)	1,719 (49.4)	0.003
Diabetes mellitus, *n* (%)	1,215 (21.2)	518 (22.9)	697 (20.0)	0.010
CVD, *n* (%)	1,291 (22.5)	447 (19.7)	844 (24.3)	<0.001
COPD, *n* (%)	376 (6.5)	147 (6.5)	229 (6.6)	0.886
Chronic kidney disease, *n* (%)	338 (5.9)	131 (5.8)	207 (6.0)	0.789
Liver disease, *n* (%)	398 (6.9)	167 (7.4)	231 (6.6)	0.288
Cancer, *n* (%)	537 (9.4)	195 (8.6)	342 (9.8)	0.119
Depression, *n* (%)	286 (5.0)	135 (6.0)	151 (4.3)	0.006
Insomnia, *n* (%)	1,041 (18.1)	374 (16.5)	667 (19.2)	0.010
Hypnotics usage, *n* (%)	594 (10.3)	209 (9.2)	385 (11.1)	0.025
Follow up, years	6.2 (4.6, 8.1)	6.1 (4.5, 8.0)	6.3 (4.6, 8.1)	0.121
PAP acceptance, %	3,094 (54.1)	1,251 (55.5)	1,843 (53.1)	0.078

Data are reported as median (IQR) values and count (%).

AHI, apnea-hypopnea index; BMI, body mass index; COPD, chronic obstructive pulmonary disease; CVD, cardiovascular disease; EDS, excessive daytime sleepiness; ESS, Epworth Sleepiness Scale; IQR, interquartile range; OSA, obstructive sleep apnea; PAP, positive airway pressure; PLMI, periodic limb movement index; RDI, respiratory disturbance index; SpO_2_, oxyhemoglobin saturation; T90, total sleep time (in percent) spent with SpO_2_ below 90%.

Numbers may not sum to totals because of missing data.

*P*-values from Mann–Whitney *U* test or Pearson Chi square test.

### 3.2. Association between sleepiness and chronic diseases

**Figure 1:**
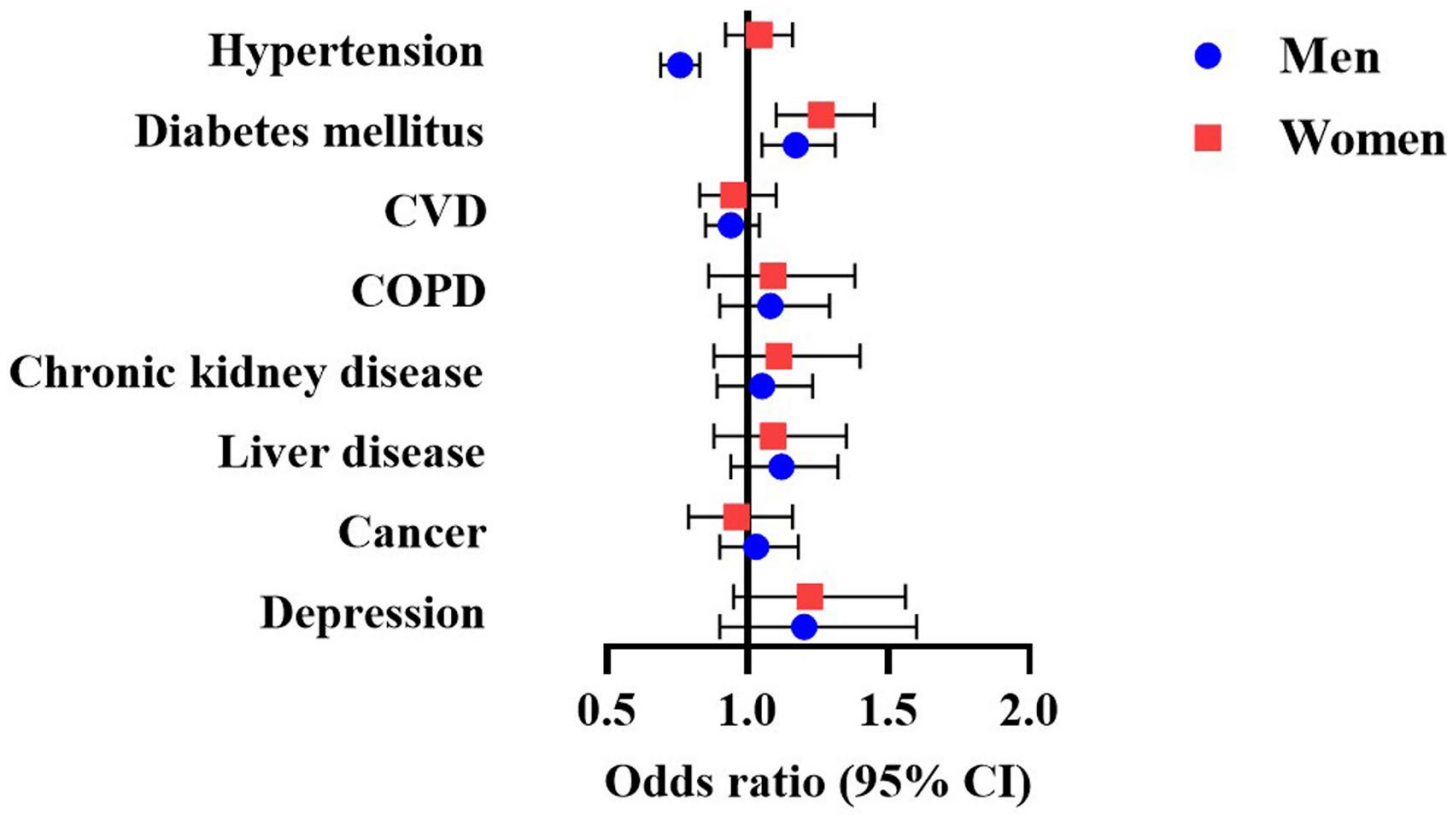
Multivariable-adjusted odds ratio (95% CI) of the association between EDS and chronic disease in men and women with OSA. Models adjusted for age, BMI, race, smoking history, AHI, T90, arousal index, sleep efficiency, PLMI, insomnia and hypnotics usage. AHI, apnea-hypopnea index; BMI, body mass index; COPD, chronic obstructive pulmonary disease; CVD, cardiovascular disease; EDS, excessive daytime sleepiness; OSA, obstructive sleep apnea; PLMI, periodic limb movement index; T90, total sleep time (in percent) spent with SpO_2_ below 90%.

Figure 1 and Supplementary Table 3 show the cross-sectional relationships between sleepiness and chronic diseases. In models adjusted for demographics, smoking history and sleep variables, odds of hypertension were lower in men with ESS > 10 than in men with ESS ≤ 10 (OR 0.76, 95% CI 0.69–0.83), while no statistically significant association was evident in women. Conversely, ESS > 10 was associated with greater risk of diabetes mellitus in both men (OR 1.17, 95% CI 1.05–1.31) and women (OR 1.26, 95% CI 1.10–1.45). Consistently, we observed a negative relationship between ESS score and hypertension only in men (OR per 1-point increase in ESS: 0.97, 95% CI 0.96–0.98), while risk of diabetes increased with increasing ESS in both men (OR 1.02, 95% CI 1.00–1.03) and women (OR 1.03, 95% CI 1.01–1.04; Supplementary Table 3). Additionally, odds for depression were 1.03-times higher (95% CI 1.01–1.06) for each 1-point increase in ESS in women. In men, there was a significant interaction between EDS and age for depression, with odds for depression being higher in sleepy men ≥65 years (Supplementary Table 4). A similar pattern was noted in men for cancer risk (P for interaction with age = 0.013), but stratified ORs did not achieve statistical significance. No other significant interactions were noted (Supplementary Tables 4–6).

Restricted cubic spline analysis showed curvilinear relations between ESS scores and diabetes in both men and women (Ps for non-linearity = 0.016 and 0.021, respectively; Supplementary Figure 2). Among men, odds for depression were elevated at both lower and higher ESS scores (P for non-linearity = 0.034). In women, risk of cancer was lower at both extremes of the score range (P for non-linearity = 0.002).

### 3.3. Association between sleepiness and mortality

Median follow-up was 6.2 (4.4, 8.1) years in men and 6.2 (4.6, 8.1) years in women, with no difference between those with and without EDS in either group (Tables 1, 2). Kaplan–Meier estimates of survival were not significantly different between sleepy vs. non-sleepy men (log-rank test, *p* = 0.596) or women (*p* = 0.996; Supplementary Figure 3). However, in Cox models adjusted for demographics and smoking history, the risk of death was 1.26-times (95% CI 1.07–1.49) greater in women with ESS > 10 vs. ESS ≤ 10 (Table 3). This association was not attenuated after further accounting for sleep characteristics and diseases at baseline. In a fully adjusted model, the HR for mortality in women with ESS > 10 compared to those with ESS ≤ 10 was 1.24 (95% CI 1.05–1.47). In men, ESS > 10 was not associated with mortality in any models. Treating ESS as continuous variable yielded consistent results, with ESS score predicting higher mortality risk in women (HR per 1-point increase in ESS: 1.03, 95% CI 1.01–1.04), but not in men. No evidence of curvilinear associations or effect modifications of age, BMI or AHI were noted (Supplementary Figure 4; Supplementary Table 7).

**Table 3:** Association between sleepiness and all-cause mortality in OSA men and women.

		HR (95% CI)		
	No. deaths	Model 1	Model 2	Model 3
Men	1,234			
ESS > 10		1.03 (0.92–1.15)	1.05 (0.94–1.19)	1.02 (0.91–1.15)
ESS, per 1-point		1.00 (0.99–1.02)	1.01 (0.99–1.02)	1.00 (0.99–1.02)
Women	595			
ESS > 10		1.26 (1.07–1.49)	1.26 (1.06–1.49)	1.24 (1.05–1.47)
ESS, per 1-point		1.03 (1.01–1.05)	1.03 (1.01–1.05)	1.03 (1.01–1.04)

Model 1 adjusted for age, BMI, race, and smoking.

Model 2 adjusted for variables included in Model 1, AHI, T90, arousal index, sleep efficiency, PLMI, insomnia and hypnotics usage.

Model 3 adjusted for variables included in Model 2, hypertension, diabetes mellitus, CVD, COPD, chronic kidney disease, liver disease, cancer, and depression.

AHI, apnea-hypopnea index; BMI, body mass index; COPD, chronic obstructive pulmonary disease; CVD, cardiovascular disease; ESS, Epworth Sleepiness Scale; OSA, obstructive sleep apnea; PLMI, periodic limb movement index; T90, total sleep time (in percent) spent with SpO_2_ below 90%.

### 3.4. Additional analyses

In sensitivity analysis (Supplementary Table 8), exclusion of deaths within 6 (HR 1.18, 95% CI 0.99–1.40) and 12 months (HR 1.17, 95% CI 0.98–1.40) from the index date attenuated the association between ESS > 10 and mortality in women. Conversely, HRs for ESS as a continuous variable did not decrease and the association with mortality remained significant in women (HR per 1-point increase in ESS:1.03, 95% CI 1.01–1.04 for both subanalyses). Similarly, excluding patients with CVD (HR 1.22, 95% CI 0.96–1.54) or cancer (HR 1.22, 95% CI 1.01–1.47) at baseline marginally diminished the strength of the relation between ESS score and mortality in women. However, ESS as a continuous variable remained again a significant predictor of death in women without CVD (HR per 1-point increase in ESS: 1.03, 95% CI 1.01–1.06) or cancer (HR per 1-point increase in ESS: 1.03, 95% CI 1.01–1.05) at study entry. Furthermore, HRs did not appreciably change after further adjusting for PAP acceptance (HR 1.25, 95% CI 1.06–1.48), nor after restricting the sample to patients who underwent split-night studies (HR 1.28, 95% CI 1.08–1.53).

Lastly, patients without ESS were older and less likely to be White than those with ESS (Supplementary Table 9). They also had higher AHI and T90 and were more likely to suffer from chronic diseases including hypertension, diabetes mellitus, CVD, COPD, and chronic kidney disease.

## 4. Discussion

In this large OSA sample, EDS measured by ESS showed sex-specific associations with multiple chronic diseases. Curvilinear relations with ESS scores suggest non-linear dynamics between degrees of perceived sleepiness and disease profiles. Furthermore, EDS was an independent predictor of all-cause mortality only in OSA women.

Consistent with prior estimates (Kapur et al., 2005; Koutsourelakis et al., 2008; Nigro et al., 2018), approximately 40% of our patients reported ESS > 10, with similar prevalence between sexes despite women having milder OSA than men. Notwithstanding comparable EDS, sex differences emerged in the relationship between EDS and morbidity. Although crude prevalences of hypertension and CVD were lower in both OSA men and women with ESS > 10 vs. ESS ≤ 10, after adjusting for demographic and sleep covariates EDS remained associated only with decreased risk of hypertension and selectively in men. Some studies have linked EDS with higher blood pressure and greater risk of hypertension especially among severe OSA patients (Kapur et al., 2008; Koutsourelakis et al., 2008; Feng et al., 2012; Ren et al., 2016), but others did not confirm this relationship (de la Peña Bravo et al., 2007; Roure et al., 2008; Prasad et al., 2018). Recent investigations show that OSA patients with normal blood pressure have higher ESS scores than OSA patients with hypertension, with the highest score reported by normotensives with moderate to severe OSA (Martynowicz et al., 2017; Tam et al., 2019). Notably, these studies included predominantly males. Although mechanisms are unclear, the lower degree of sleepiness in those with comorbid OSA and hypertension may be a manifestation of hyperarousal (Tam et al., 2019). Co-occurrence of diseases characterized by sympathetic hyperactivation could offset OSA-induced sleepiness by stimulating alertness *via* central adrenergic over-excitation. Such effect could be more evident in men due to their relatively higher basal adrenergic tone (Hart et al., 2012).

Conversely, multivariable-adjusted odds for diabetes mellitus were greater in both sleepy men and women relative to their non-sleepy counterparts. Sleepiness is associated with poor health and worse glycemic control among diabetic patients with (Barceló et al., 2008; Aurora and Punjabi, 2019) and without OSA (Chasens et al., 2009; Inkster et al., 2013). EDS predicts diabetes independently of OSA symptoms (Lindberg et al., 2007), and modifies the relationship between OSA and diabetes, with only sleepy OSA patients exhibiting greater risk of diabetes (Ronksley et al., 2009). With respect to biological pathways, hypersomnolence has been associated with increased inflammation, a known precursor of insulin resistance and beta-cell dysfunction, in some (Vgontzas et al., 1997; Li et al., 2017) but not all studies (de la Peña Bravo et al., 2007; Dixon et al., 2007). Decreased cerebral glucose uptake, as observed after sleep deprivation (Wu et al., 2006), may also contribute to both sleepiness and dysregulated glucose metabolism (Tasali et al., 2009).

Modeling the ESS score as a continuous variable unmasked further independent effects, such as on the risk of depression. While female sex and EDS were predictors of depression in OSA participants of the Penn State Adult cohort (LaGrotte et al., 2016), Basta et al. (2008) found that depression was more closely associated with severe sleepiness in men than in women. Our results may aid in reconciling these discrepancies, showing that higher ratings of sleepiness were linearly associated with depression in women, while a curvilinear relation was noted among men. On the other hand, an inverse U-shaped association between ESS and cancer was apparent in women, with reduced risk of cancer at both lower and higher ESS scores. Interestingly, Ohayon et al. (Ohayon, 2012) found a significant association between cancer and moderate sleepiness but not severe sleepiness. In aggregate, these findings illustrate not only distinct patterns of disease profile in sleepy OSA men and women, but also indicate that these risk configurations are partly determined by the definition of pathological sleepiness, thus shedding light into prior conflicting evidence.

A robust sex-dependent pattern emerged also for mortality risk, with ESS independently predicting greater risk of death only in OSA women. Notably, this association emerged only after taking into account demographic characteristics, suggesting that these are important sex-specific confounders in the relation between sleepiness and mortality. Differences in the relationship between EDS and mortality as a function of sex have been noted in general population studies, albeit with discordant findings (Newman et al., 2000; Empana et al., 2009; Boden-Albala et al., 2012; Li et al., 2021). With regard to OSA, sleepiness did not modify the increased mortality risk exhibited by severe OSA participants of the Wisconsin sleep cohort (Young et al., 2008), while the excessively sleepy OSA phenotype manifested the worst survival among OSA symptom subtypes from the Sleep Heart Health Study (Mazzotti et al., 2019) – although no sex-stratified data were reported in either study. A recent study on patients with suspected OSA found that EDS predicted a composite CV endpoint (including all-cause deaths) only in women (Kendzerska et al., 2020). These data are consistent with our results on patients with confirmed OSA, and corroborate the concept that hypersomnolence may be an independent predictor of mortality in women.

The reasons underlying this observation remain unclear, and unmeasured factors may be involved. Among these, growing evidence implicates insufficient sleep as a contributor to increased morbidity and mortality (Covassin and Singh, 2016; Itani et al., 2017). However, studies on differences in habitual sleep duration between sleepy and non-sleepy individuals are mixed, including among OSA (Kapur et al., 2005; Koutsourelakis et al., 2008; Prasad et al., 2018). Because excluding early deaths attenuated the association between ESS > 10 and mortality in women, sleepiness could be an indicator of subclinical diseases or overall poor health status. Nevertheless, estimates were unaltered when using continuous ESS, suggesting limited confounding influence of any fatal undiagnosed conditions.

Mechanistically, although women with OSA have higher levels of inflammatory markers (Gaines et al., 2015), the inflammatory burden is not associated with sleepiness among them (Svensson et al., 2012). Kritikou et al. (2014) noted instead a stronger association between sleepiness and inflammation in OSA men. Other candidates plausibly implicated in our results include increased oxidative stress and vascular dysfunction (Wang et al., 2015; Lira and de Sousa Rodrigues, 2016). However, data on sex differences on these potential mechanisms are scarce and inconclusive, as is their relation with sleepiness in OSA (de la Peña Bravo et al., 2007; Šiarnik et al., 2014). Further research targeting the pathophysiological pathways underlying the heightened risk of death in sleepy OSA women is warranted.

Our study has important clinical implications. Because our study shows that OSA men with hypertension were less likely to report EDS, a negative ESS should not be used to rule out OSA among hypertensive patients. On the other hand, an elevated ESS is a sentinel for diabetes in both sexes, thus underscoring the need to consider screening for diabetes in those complaining of hypersomnolence. From a therapeutic perspective, despite OSA therapy improves EDS (Patil et al., 2019), residual sleepiness persists in a substantial proportion of treated patients. In our study, while OSA treatment could have attenuated the strength of the relation between sleepiness and mortality by ameliorating EDS, further adjusting for PAP acceptance did not affect the results. Although only crude information on therapy was available, it is possible that suboptimal resolution of EDS in OSA women may be implicated in their survival disadvantage.

A strength of our study is its large sample size enabling adequate female representation. Sleepiness was determined by ESS, a validated instrument broadly used to quantify symptoms of sleepiness, thus enhancing applicability of our results. On the other hand, the ESS is inherently prone to recall bias and misperception, and additional studies including objective sleepiness measures are needed. As the relation between EDS and chronic diseases was assessed cross-sectionally, causality cannot be determined. Generalizability may be limited as our sample comprises mostly of White individuals and was drawn from the patient population evaluated at a sleep clinic, and thus subject to referral bias. Because the acceptable definition of hypopnea (i.e., 4% oxygen desaturation scoring standard) was applied when scoring respiratory events, this likely led to lower estimates of OSA severity than if the recommended standard (i.e., 3% desaturation or arousal) were used instead. Last, as the near totality of the sample underwent split-night studies, evaluation of OSA features associated with sleep architecture could not be performed.

In summary, our study shows sex-specific patterns of associations between perceived sleepiness and chronic disease and mortality in OSA patients. Recognizing that the predictive value of EDS is sex-dependent is critical to better understanding its health implications and to develop targeted therapeutic approaches.

## Data availability statement

The data analyzed in this study is subject to the following licenses/restrictions: Deidentified participant’s data will be available for scientific research upon request submitted to the corresponding author. Requests will be reviewed for suitability. Data will be made available providing IRB approval and a data sharing agreement, in accordance with data sharing policies and Mayo Clinic IRB requirements, are obtained. Requests to access these datasets should be directed to somers.virend@mayo.edu.

## Ethics statement

The studies involving human participants were reviewed and approved by Mayo Clinic Institutional Review Board. The ethics committee waived the requirement of written informed consent for participation.

## Author contributions

NC, DL, AC, ES, and VS conceived and designed the study. NC, DL, PS, and JL analyzed the data. NC, DL, ES, and VS drafted the manuscript. NC, DL, and VS have direct access and verified the data. All authors revised the manuscript for important intellectual content and approved the final version of the manuscript.

## Funding

Financial support to this study was provided by National Institutes of Health grants RO1 HL114676 and RO1 HL65176 and by a grant from Sleep Number to Mayo Clinic for studies of sleepiness. The contents of this article are solely the responsibility of the authors and do not necessarily represent the official view of the NIH. The funders of the study had no role in study design, data collection, data analysis, data interpretation, or writing of the report.

## Conflict of interest

EO is a member of the Board of Directors of the American Academy of Sleep Medicine. VS serves as a consultant for ResMed, Jazz Pharmaceuticals, Bayer, Lilly, Sleep Number, Zoll, Respicardia, and Huxley.

The remaining authors declare that the research was conducted in the absence of any commercial or financial relationships that could be construed as a potential conflict of interest.

## Publisher’s note

All claims expressed in this article are solely those of the authors and do not necessarily represent those of their affiliated organizations, or those of the publisher, the editors and the reviewers. Any product that may be evaluated in this article, or claim that may be made by its manufacturer, is not guaranteed or endorsed by the publisher.
