# Supplementary material for: Sex-specific associations between daytime sleepiness, chronic diseases and mortality in obstructive sleep apnea
Source: Front Neurosci. 2023 Jun 23;17:1210206. doi: 10.3389/fnins.2023.1210206 (PMC10326268; doi:10.3389/fnins.2023.1210206)
Supplement: Supplementary file 1 [file Data_Sheet_1.docx]

**Supplementary Material**

**Supplementary Figure 1. Patient flowchart.**

**
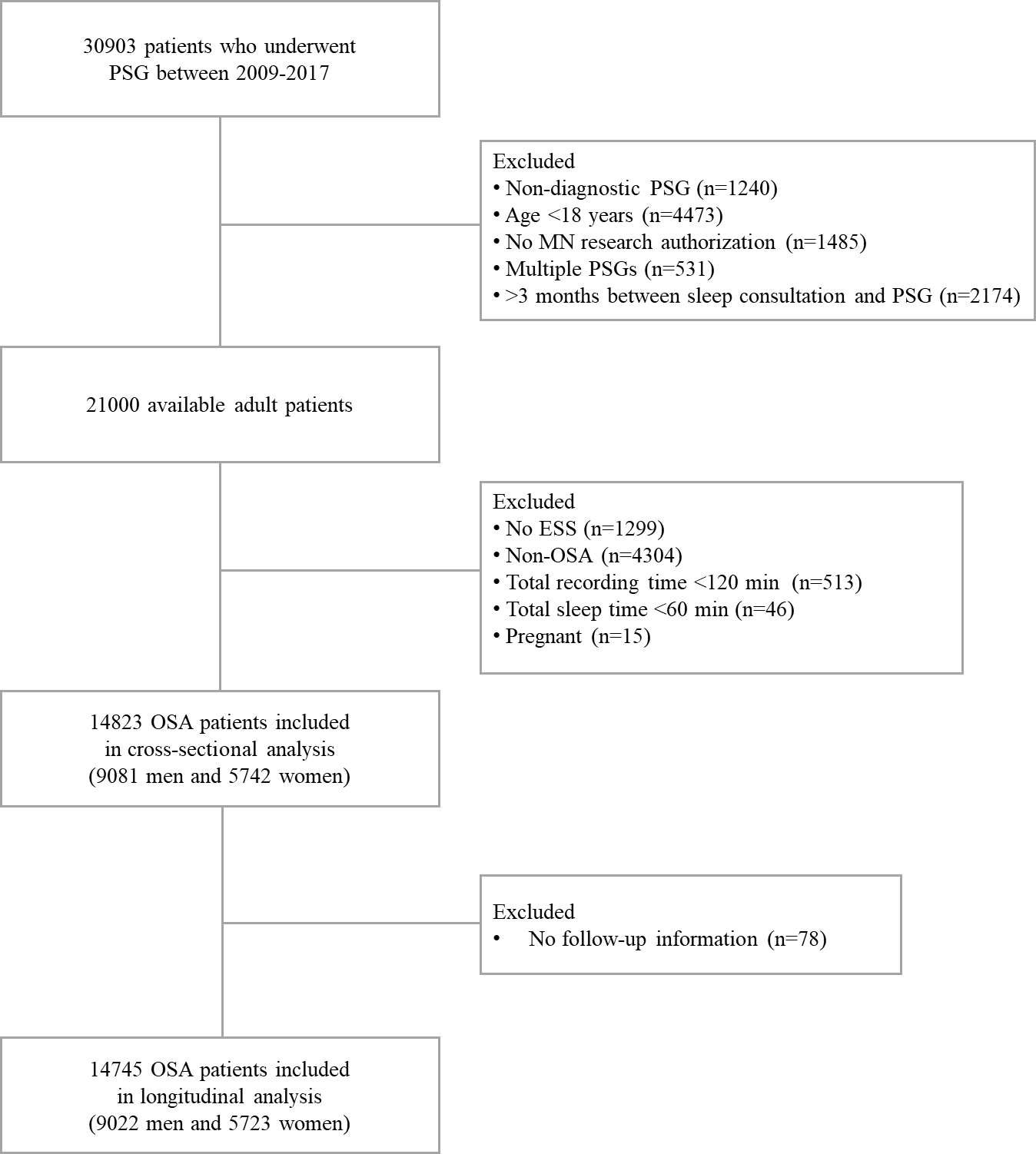
**

**Supplementary Figure 2. Multivariable-adjusted odds ratio (95% CI) of the association between ESS score and chronic disease in men and women with OSA.** Men are depicted in blue, women in red. Models adjusted for age, BMI, race, smoking history, AHI, T90, arousal index, sleep efficiency, PLMI, insomnia and hypnotics usage. ESS = 11 was used as a reference. AHI, apnea-hypopnea index; BMI, body mass index; COPD, chronic obstructive pulmonary disease; CVD, cardiovascular disease; ESS, Epworth Sleepiness Scale; OSA, obstructive sleep apnea; PLMI, periodic limb movement index; T90, total sleep time (in percent) spent with SpO2 below 90%.


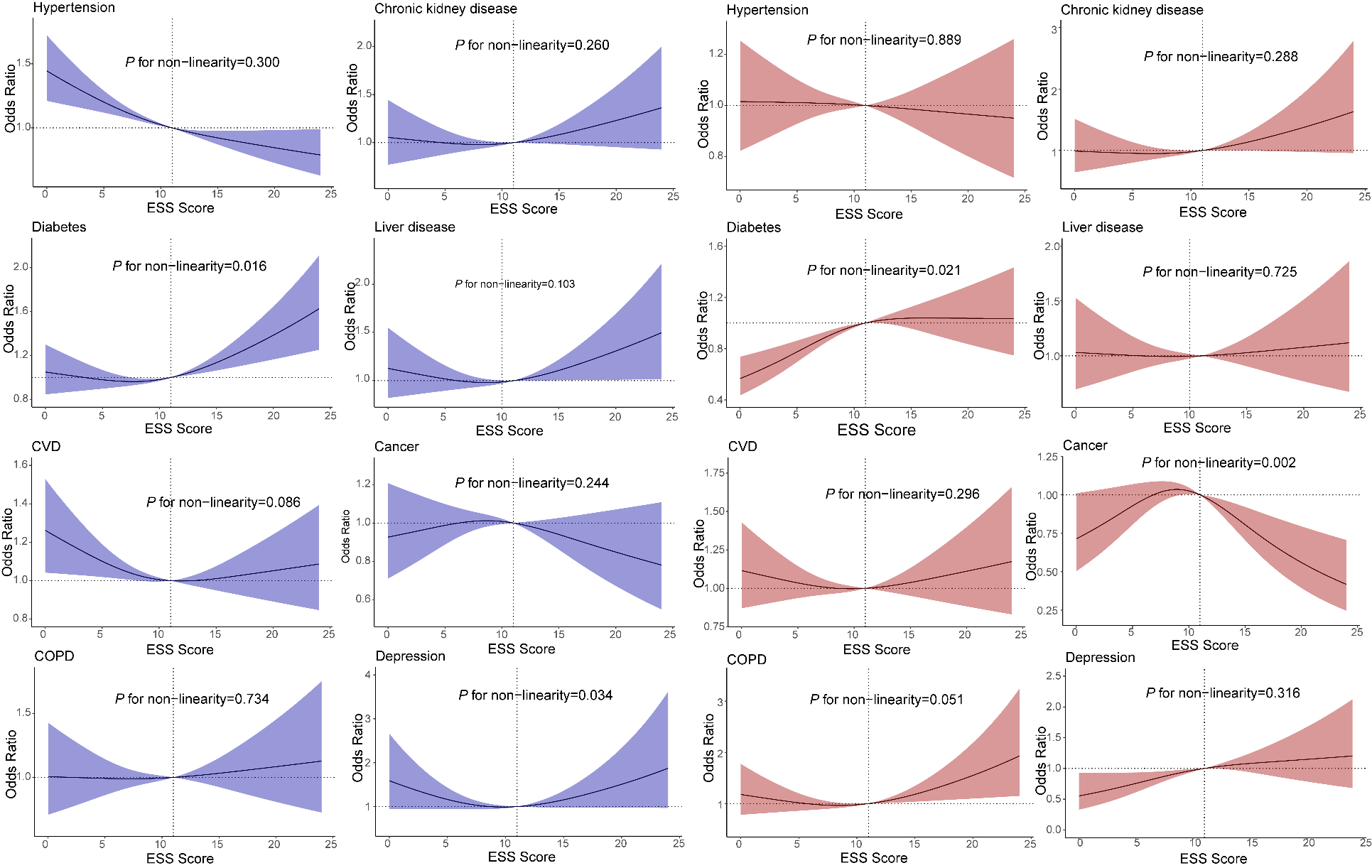


**Supplementary Figure 3. Kaplan-Meier estimates of survival in sleepy (ESS > 10) vs non-sleepy (ESS ≤ 10) men and women with OSA.** Dotted lines are 95% CI. ESS, Epworth Sleepiness Scale; OSA, obstructive sleep apnea.

**Supplementary Figure 4. Multivariable-adjusted hazard ratio (95% CI) of the association between ESS score and all-cause mortality in men and women with OSA.** Men are depicted in blue, women in red. Models adjusted for age, BMI, race, smoking history, AHI, T90, arousal index, sleep efficiency, PLMI, insomnia, hypnotics usage, hypertension, diabetes mellitus, CVD, COPD, chronic kidney disease, liver disease, cancer, and depression. ESS = 11 was used as reference. AHI, apnea-hypopnea index; BMI, body mass index; COPD, chronic obstructive pulmonary disease; CVD, cardiovascular disease; ESS, Epworth Sleepiness Scale; OSA, obstructive sleep apnea; PLMI, periodic limb movement index; T90, total sleep time (in percent) spent with SpO2 below 90%.


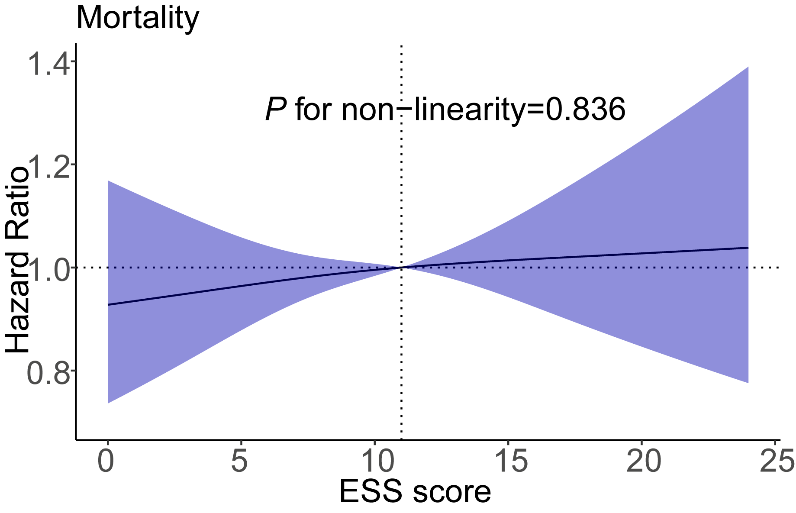

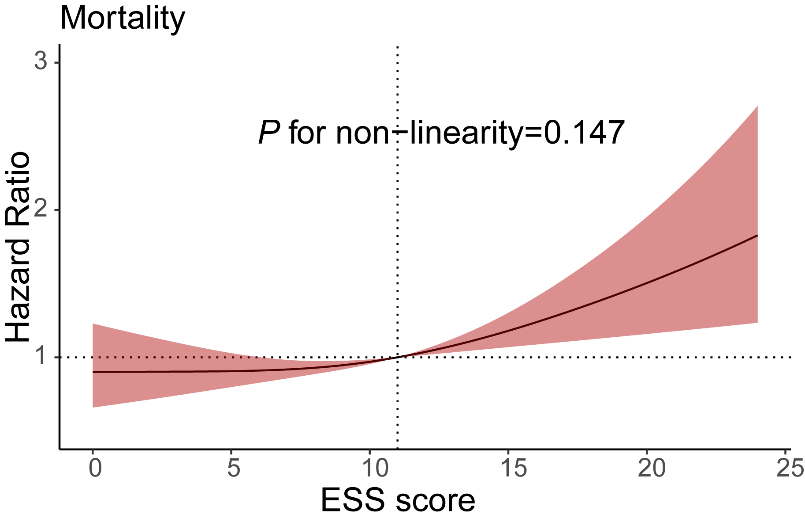


**Supplementary Table 1. List of International Classification of Diseases codes.**

| **Variable** | **ICD-9 codes** | **ICD-10 codes** |
| --- | --- | --- |
| Hypertension | 401-405 | I10-I15 |
| Diabetes mellitus | 249-250 | E10-E14 |
| CVD | 390-459 | I00-I99 |
| COPD | 491-492, 496 | J41-J44 |
| Chronic kidney disease | 585 | N18 |
| Liver disease | 570-573 | K70-K77 |
| Cancer | 140-209 | C00-C97 |
| Depression | 296.2, 296.3, 300.4, 311 | F32-F34.1, F38.1 |
| Insomnia | 307.42, 307.49, 327.0, 780.51, 780.52 | F51.01, F51.04, F51.09, G47.0 |

ICD, International Classification of Diseases; COPD, chronic obstructive pulmonary disease; CVD, cardiovascular disease.

**Supplementary Table 2. Characteristics of men and women with OSA.**

| **Characteristic** | **Total**  **(n = 14,823)** | **Men**  **(n = 9,081)** | **Women**  **(n = 5,742)** | **P value** |
| --- | --- | --- | --- | --- |
| Age, years | 61 (51, 70) | 60 (50, 70) | 61 (51, 70) | 0.067 |
| White, n (%) | 13,504 (92.7) | 8,264 (92.8) | 5,240 (92.4) | 0.347 |
| BMI, kg/m^2^ | 32.6 (28.7, 37.6) | 31.9 (28.5, 35.9) | 34.2 (29.3, 40.2) | < 0.001 |
| Smoking history, n (%) | 4,589 (31.2) | 3,031 (33.6) | 1,558 (27.3) | < 0.001 |
| Sleep measures |  |  |  |  |
| ESS score | 9 (5, 13) | 9 (5, 13) | 9 (5, 13) | 0.007 |
| ESS > 10 | 5,912 (39.9) | 3,647 (40.2) | 2,265 (39.4) | 0.387 |
| Split-night, n (%) | 14,039 (94.7) | 8,642 (95.2) | 5,397 (94.0) | 0.002 |
| Total recording time, min | 229 (187, 281) | 222 (182, 272) | 240 (196, 292) | < 0.001 |
| Total sleep time, min | 158 (132, 194) | 153 (129, 183) | 168 (139, 209) | < 0.001 |
| Sleep efficiency, % | 75.5 (62.5, 85.1) | 74.9 (61.8, 84.6) | 76.5 (63.7, 85.7) | < 0.001 |
| Arousal index, events/h | 35.9 (23.6, 55.0) | 39.2 (26.1, 59.3) | 31.3 (20.1, 47.9) | < 0.001 |
| PLMI, events/h | 15.3 (0.0, 52.8) | 18.3 (0.9, 60.2) | 11.1 (0.0, 42.5) | < 0.001 |
| AHI, events/h | 17 (8, 35) | 20 (10, 40) | 13 (7, 26) | < 0.001 |
| RDI, events/h | 27 (15, 48) | 30 (17, 53) | 22 (13, 39) | < 0.001 |
| Mean SpO_2_, % | 93 (91, 94) | 93 (91, 94) | 93 (91, 94) | 0.007 |
| Minimum SpO_2_, % | 83 (78, 86) | 83 (78, 86) | 83 (78, 86) | 0.781 |
| T90, % | 4.7 (1.1, 17.5) | 5.1 (1.3, 17.6) | 4.0 (1.0, 17.5) | 0.001 |
| OSA severity |  |  |  | < 0.001 |
| AHI 5-14.9, n (%) | 6,624 (44.7) | 3,500 (38.5) | 3,124 (54.4) |  |
| AHI 15-29.9, n (%) | 3,714 (25.1) | 2,353 (25.9) | 1,361 (23.7) |  |
| AHI ≥ 30, n (%) | 4,485 (30.3) | 3,228 (35.5) | 1,257 (21.9) |  |
| Comorbidities |  |  |  |  |
| Hypertension, n (%) | 7,157 (48.3) | 4,410 (48.6) | 2,747 (47.8) | 0.391 |
| Diabetes mellitus, n (%) | 3,083 (20.8) | 1,868 (20.6) | 1,215 (21.2) | 0.389 |
| CVD, n (%) | 4,292 (29.0) | 3,001 (33.0) | 1,291 (22.5) | < 0.001 |
| COPD, n (%) | 1,015 (6.8) | 639 (7.0) | 376 (6.5) | 0.251 |
| Chronic kidney disease, n (%) | 1,071 (7.2) | 733 (8.1) | 338 (5.9) | < 0.001 |
| Liver disease, n (%) | 1,042 (7.0) | 644 (7.1) | 398 (6.9) | 0.710 |
| Cancer, n (%) | 1,634 (11.0) | 1097 (12.1) | 537 (9.4) | < 0.001 |
| Depression, n (%) | 495 (3.3) | 209 (2.3) | 286 (5.0) | < 0.001 |
| Insomnia, n (%) | 2,049 (13.8) | 1,008 (11.1) | 1,041 (18.1) | < 0.001 |
| Hypnotics usage, n (%) | 1,384 (9.3) | 790 (8.7) | 594 (10.3) | 0.001 |
| Follow up, years | 6.2 (4.5, 8.1) | 6.2 (4.4, 8.1) | 6.2 (4.6, 8.1) | 0.292 |
| PAP acceptance, % | 8,003 (54.3) | 4,909 (54.4) | 3,094 (54.1) | 0.679 |

Data are reported as median (IQR) values and count (%).

Abbreviations: AHI, apnea-hypopnea index; BMI, body mass index; COPD, chronic obstructive pulmonary disease; CVD, cardiovascular disease; ESS, Epworth Sleepiness Scale; IQR, interquartile range; OSA, obstructive sleep apnea; PAP, positive airway pressure; PLMI, periodic limb movement index; RDI, respiratory disturbance index; SpO_2_, oxyhemoglobin saturation; T90, total sleep time (in percent) spent with SpO_2_ below 90%.

Numbers may not sum to totals because of missing data.

P-values from Mann-Whitney U test or Pearson Chi square test.

**Supplementary Table 3 Association between sleepiness and chronic diseases in men and women with OSA.**

|  | **Men** | | **Women** | |
| --- | --- | --- | --- | --- |
|  | Model 1 | Model 2 | Model 1 | Model 2 |
|  | OR (95% CI) | OR (95% CI) | OR (95% CI) | OR (95% CI) |
| **Hypertension** |  |  |  |  |
| ESS > 10 | 0.75 (0.68-0.82) | 0.76 (0.69-0.83) | 1.03 (0.92-1.16) | 1.04 (0.92-1.16) |
| ESS, per 1-point | 0.97 (0.96-0.98) | 0.97 (0.96-0.98) | 1.00 (0.99-1.01) | 1.00 (0.99-1.01) |
| **Diabetes mellitus** |  |  |  |  |
| ESS > 10 | 1.16 (1.04-1.30) | 1.17 (1.05-1.31) | 1.27 (1.11-1.45) | 1.26 (1.10-1.45) |
| ESS, per 1-point | 1.01 (1.00-1.03) | 1.02 (1.00-1.03) | 1.03 (1.02-1.04) | 1.03 (1.01-1.04) |
| **CVD** |  |  |  |  |
| ESS > 10 | 0.91 (0.83-1.01) | 0.94 (0.85-1.04) | 0.94 (0.82-1.08) | 0.95 (0.83-1.10) |
| ESS, per 1-point | 0.99 (0.98-1.00) | 0.99 (0.98-1.00) | 1.00 (0.98-1.01) | 1.00 (0.99-1.01) |
| **COPD** |  |  |  |  |
| ESS > 10 | 1.01 (0.85-1.20) | 1.08 (0.90-1.29) | 1.09 (0.87-1.37) | 1.09 (0.86-1.38) |
| ESS, per 1-point | 1.00 (0.99-1.02) | 1.01 (0.99-1.03) | 1.01 (0.99-1.03) | 1.02 (0.99-1.04) |
| **Chronic kidney disease** |  |  |  |  |
| ESS > 10 | 1.03 (0.88-1.21) | 1.05 (0.89-1.23) | 1.12 (0.89-1.41) | 1.11 (0.88-1.40) |
| ESS, per 1-point | 1.00 (0.99-1.02) | 1.01 (0.99-1.02) | 1.02 (1.00-1.04) | 1.02 (1.00-1.04) |
| **Liver disease** |  |  |  |  |
| ESS > 10 | 1.07 (0.91-1.26) | 1.12 (0.94-1.32) | 1.06 (0.86-1.31) | 1.09 (0.88-1.35) |
| ESS, per 1-point | 1.00 (0.99-1.02) | 1.01 (0.99-1.03) | 1.00 (0.98-1.02) | 1.00 (0.98-1.02) |
| **Cancer** |  |  |  |  |
| ESS > 10 | 1.03 (0.90-1.17) | 1.03 (0.90-1.18) | 0.96 (0.79-1.15) | 0.96 (0.79-1.16) |
| ESS, per 1-point | 1.00 (0.98-1.01) | 1.00 (0.98-1.01) | 0.99 (0.97-1.01) | 0.99 (0.97-1.01) |
| **Depression** |  |  |  |  |
| ESS > 10 | 1.11 (0.84-1.47) | 1.20 (0.90-1.60) | 1.16 (0.91-1.49) | 1.22 (0.95-1.56) |
| ESS, per 1-point | 1.00 (0.97-1.02) | 1.00 (0.98-1.03) | 1.03 (1.00-1.05) | 1.03 (1.01-1.06) |

Model 1 adjusted for age, BMI, race, and smoking history.

Model 2 adjusted for variables included in Model 1, and AHI, T90, arousal index, sleep efficiency, PLMI, insomnia and hypnotics usage.

AHI, apnea-hypopnea index; BMI, body mass index; COPD, chronic obstructive pulmonary disease; CVD, cardiovascular disease; ESS, Epworth Sleepiness Scale; OSA, obstructive sleep apnea; PLMI, periodic limb movement index; T90, total sleep time (in percent) spent with SpO_2_ below 90%.

**Supplementary Table 4. Association between EDS (ESS > 10) and chronic diseases in men and women with OSA stratified by age.**

|  | **Men** | | | **Women** | | |
| --- | --- | --- | --- | --- | --- | --- |
|  | Age < 65  (n=5,295) | Age ≥ 65  (n=3,536) | *P-interaction* | Age < 65  (n=3,365) | Age ≥ 65  (n=2,253) | *P-interaction* |
| Hypertension | 0.74 (0.66-0.83) | 0.79 (0.68-0.91) | 0.766 | 1.03 (0.90-1.19) | 0.88 (0.73-1.05) | 0.089 |
| Diabetes mellitus | 1.15 (0.98-1.34) | 1.17 (1.00-1.37) | 0.262 | 1.19 (1.00-1.43) | 1.25 (1.01-1.55) | 0.530 |
| CVD | 0.88 (0.76-1.01) | 0.98 (0.85-1.12) | 0.623 | 0.93 (0.76-1.14) | 0.88 (0.73-1.06) | 0.220 |
| COPD | 1.00 (0.74-1.34) | 1.12 (0.90-1.41) | 0.833 | 1.02 (0.72-1.44) | 1.08 (0.78-1.49) | 0.210 |
| Chronic kidney disease | 1.06 (0.81-1.37) | 1.05 (0.86-1.29) | 0.974 | 1.10 (0.76-1.57) | 1.09 (0.80-1.49) | 0.934 |
| Liver disease | 1.03 (0.84-1.27) | 1.33 (0.99-1.77) | 0.180 | 0.94 (0.72-1.21) | 1.49 (1.04-2.14) | 0.209 |
| Cancer | 0.87 (0.70-1.09) | 1.15 (0.97-1.37) | 0.013 | 0.97 (0.75-1.26) | 0.89 (0.67-1.17) | 0.673 |
| Depression | 0.99 (0.71-1.38) | 2.37 (1.29-4.36) | 0.016 | 1.31 (0.99-1.72) | 1.30 (0.74-2.27) | 0.714 |

Estimates are reported as odds ratio and 95% CI.

Models adjusted for BMI, race, smoking history, AHI, T90, arousal index, sleep efficiency, PLMI, insomnia and hypnotics usage.

AHI, apnea-hypopnea index; BMI, body mass index; COPD, chronic obstructive pulmonary disease; CVD, cardiovascular disease; EDS, excessive daytime sleepiness; ESS, Epworth Sleepiness Scale; OSA, obstructive sleep apnea; PLMI, periodic limb movement index; T90, total sleep time (in percent) spent with SpO_2_ below 90%.

**Supplementary Table 5. Association between EDS (ESS > 10) and chronic diseases in men and women with OSA stratified by BMI.**

|  | **Men** | | | **Women** | | |
| --- | --- | --- | --- | --- | --- | --- |
|  | BMI < 30 (n=3,126) | BMI ≥ 30 (n=5,705) | *P-interaction* | BMI < 30 (n=1,618) | BMI ≥ 30 (n=4,000) | *P-interaction* |
| Hypertension | 0.75 (0.64-0.88) | 0.76 (0.68-0.85) | 0.340 | 0.92 (0.73-1.16) | 1.06 (0.93-1.21) | 0.605 |
| Diabetes mellitus | 1.18 (0.96-1.46) | 1.15 (1.01-1.31) | 0.835 | 1.31 (0.96-1.79) | 1.22 (1.05-1.42) | 0.143 |
| CVD | 0.91 (0.77-1.08) | 0.94 (0.83-1.07) | 0.935 | 0.96 (0.74-1.25) | 0.95 (0.80-1.13) | 0.942 |
| COPD | 1.08 (0.79-1.47) | 1.08 (0.87-1.36) | 0.420 | 1.07 (0.68-1.66) | 1.10 (0.83-1.46) | 0.928 |
| Chronic kidney disease | 0.84 (0.64-1.11) | 1.17 (0.95-1.42) | 0.917 | 1.08 (0.70-1.67) | 1.12 (0.85-1.49) | 0.919 |
| Liver disease | 0.98 (0.72-1.35) | 1.17 (0.96-1.42) | 0.092 | 0.91 (0.57-1.46) | 1.14 (0.89-1.44) | 0.116 |
| Cancer | 0.99 (0.81-1.22) | 1.07 (0.89-1.27) | 0.626 | 0.90 (0.64-1.27) | 0.99 (0.79-1.25) | 0.949 |
| Depression | 1.22 (0.77-1.95) | 1.18 (0.82-1.71) | 0.678 | 1.16 (0.68-1.98) | 1.23 (0.93-1.64) | 0.851 |

Estimates are reported as odds ratio and 95% CI.

Models adjusted for age, race, smoking history, AHI, T90, arousal index, sleep efficiency, PLMI, insomnia and hypnotics usage.

AHI, apnea-hypopnea index; BMI, body mass index; COPD, chronic obstructive pulmonary disease; CVD, cardiovascular disease; EDS, excessive daytime sleepiness; ESS, Epworth Sleepiness Scale; OSA, obstructive sleep apnea; PLMI, periodic limb movement index; T90, total sleep time (in percent) spent with SpO_2_ below 90%.

**Supplementary Table 6. Association between EDS (ESS > 10) and chronic diseases in men and women with OSA stratified by OSA severity.**

|  | **Men** | | | | **Women** | | | |
| --- | --- | --- | --- | --- | --- | --- | --- | --- |
|  | AHI 5-14.9 (n=3,393) | AHI 15-29.9  (n=2,310) | AHI ≥ 30  (n=3,128) | *P-interaction* | AHI 5-14.9  (n=3,062) | AHI 15-29.9  (n=1,326) | AHI ≥ 30  (n=1,230) | *P-interaction* |
| Hypertension | 0.72 (0.62-0.84) | 0.71 (0.59-0.85) | 0.85 (0.73-0.99) | 0.172 | 1.04 (0.89-1.22) | 1.16 (0.91-1.47) | 0.90 (0.70-1.14) | 0.237 |
| Diabetes mellitus | 1.22 (1.00-1.48) | 1.06 (0.85-1.32) | 1.23 (1.03-1.45) | 0.655 | 1.18 (0.97-1.44) | 1.50 (1.14-1.98) | 1.21 (0.92-1.57) | 0.714 |
| CVD | 0.92 (0.77-1.09) | 0.86 (0.71-1.04) | 1.02 (0.86-1.20) | 0.500 | 1.04 (0.86-1.27) | 0.82 (0.61-1.10) | 0.91 (0.68-1.22) | 0.232 |
| COPD | 1.09 (0.79-1.49) | 1.06 (0.74-1.52) | 1.04 (0.79-1.38) | 0.636 | 1.23 (0.89-1.70) | 1.11 (0.66-1.88) | 0.81 (0.51-1.30) | 0.774 |
| Chronic kidney disease | 1.00 (0.73-1.35) | 0.89 (0.65-1.20) | 1.22 (0.96-1.57) | 0.502 | 1.30 (0.92-1.85) | 1.29 (0.79-2.12) | 0.83 (0.55-1.26) | 0.226 |
| Liver disease | 1.07 (0.82-1.40) | 1.03 (0.73-1.45) | 1.26 (0.95-1.66) | 0.089 | 1.13 (0.85-1.51) | 1.09 (0.70-1.69) | 1.01 (0.64-1.60) | 0.284 |
| Cancer | 0.87 (0.70-1.10) | 1.02 (0.78-1.34) | 1.21 (0.97-1.50) | 0.264 | 0.94 (0.71-1.23) | 1.07 (0.73-1.57) | 0.88 (0.60-1.27) | 0.818 |
| Depression | 1.33 (0.85-2.09) | 0.98 (0.55-1.75) | 1.22 (0.75-2.00) | 0.239 | 1.20 (0.87-1.65) | 1.68 (0.96-2.94) | 0.92 (0.51-1.65) | 0.484 |

Estimates are reported as odds ratio and 95% CI.

Models adjusted for age, BMI, race, smoking history, T90, arousal index, sleep efficiency, PLMI, insomnia and hypnotics usage.

AHI, apnea-hypopnea index; BMI, body mass index; COPD, chronic obstructive pulmonary disease; CVD, cardiovascular disease; EDS, excessive daytime sleepiness; ESS, Epworth Sleepiness Scale; OSA, obstructive sleep apnea; PLMI, periodic limb movement index; T90, total sleep time (in percent) spent with SpO_2_ below 90%.

**Supplementary Table 7. Association between sleepiness and all-cause mortality in men and women with OSA in subgroup analysis.**

|  | **Men** | | | **Women** | | |
| --- | --- | --- | --- | --- | --- | --- |
|  | No. deaths | ESS > 10  HR (95% CI) | ESS  HR (95% CI) | No. deaths | ESS > 10  HR (95% CI) | ESS  HR (95% CI) |
| **Age** |  |  |  |  |  |  |
| < 65 years | 311 | 0.99 (0.79-1.25) | 1.00 (0.98-1.02) | 155 | 1.25 (0.91-1.72) | 1.01 (0.98-1.05) |
| ≥ 65 years | 923 | 1.05 (0.92-1.20) | 1.01 (0.99-1.02) | 440 | 1.19 (0.98-1.45) | 1.03 (1.01-1.05) |
| *P-interaction* |  | 0.454 | 0.112 |  | 0.588 | 0.691 |
| **BMI** |  |  |  |  |  |  |
| < 30 kg/m^2^ | 565 | 0.96 (0.80-1.14) | 1.00 (0.98-1.01) | 237 | 1.36 (1.04-1.78) | 1.04 (1.01-1.06) |
| ≥ 30 kg/m^2^ | 669 | 1.09 (0.93-1.27) | 1.01 (0.99-1.03) | 358 | 1.21 (0.97-1.50) | 1.03 (1.01-1.05) |
| *P-interaction* |  | 0.687 | 0.418 |  | 0.165 | 0.299 |
| **OSA severity** |  |  |  |  |  |  |
| AHI 5-14.9 | 364 | 1.06 (0.85-1.31) | 1.01 (0.99-1.03) | 265 | 1.16 (0.90-1.50) | 1.03 (1.00-1.05) |
| AHI 15-29.9 | 327 | 1.05 (0.84-1.33) | 1.02 (0.99-1.04) | 139 | 1.51 (1.06-2.15) | 1.06 (1.02-1.09) |
| AHI ≥ 30 | 543 | 1.01 (0.85-1.20) | 1.00 (0.98-1.02) | 191 | 1.22 (0.91-1.66) | 1.01 (0.98-1.05) |
| *P-interaction* |  | 0.712 | 0.846 |  | 0.216 | 0.057 |

Models adjusted for age (where appropriate), BMI (where appropriate), race, smoking, AHI (where appropriate), T90, arousal index, sleep efficiency, PLMI, insomnia, hypnotics usage, hypertension, diabetes mellitus, CVD, COPD, chronic kidney disease, liver disease, cancer, and depression.

AHI, apnea-hypopnea index; BMI, body mass index; COPD, chronic obstructive pulmonary disease; CVD, cardiovascular disease; ESS, Epworth Sleepiness Scale; OSA, obstructive sleep apnea; PLMI, periodic limb movement index; T90, total sleep time (in percent) spent with SpO_2_ below 90%.

**Supplementary Table 8. Sensitivity analyses on the association between sleepiness and all-cause mortality in men and women with OSA.**

|  | **Men** | | | **Women** | | |
| --- | --- | --- | --- | --- | --- | --- |
|  | No. deaths | ESS > 10  HR (95% CI) | ESS  HR (95% CI) | No. deaths | ESS > 10  HR (95% CI) | ESS  HR (95% CI) |
| Exclusion of deaths within 6 months | 1,155 | 0.99 (0.88-1.12) | 1.00 (0.99-1.01) | 559 | 1.18 (0.99-1.40) | 1.03 (1.01-1.04) |
| Exclusion of deaths within 12 months | 1,071 | 0.96 (0.85-1.09) | 1.00 (0.99-1.01) | 528 | 1.17 (0.98-1.40) | 1.03 (1.01-1.04) |
| Exclusion of CVD at baseline | 494 | 1.00 (0.83-1.20) | 1.00 (0.98-1.02) | 301 | 1.22 (0.96-1.54) | 1.03 (1.01-1.06) |
| Exclusion of cancer at baseline | 916 | 1.02 (0.89-1.17) | 1.01 (0.99-1.02) | 486 | 1.22 (1.01-1.47) | 1.03 (1.01-1.05) |
| PAP-adjusted model^a^ | 1,234 | 1.02 (0.91-1.15) | 1.00 (0.99-1.02) | 595 | 1.25 (1.06-1.48) | 1.03 (1.01-1.04) |
| Split-night studies only | 1,142 | 1.03 (0.91-1.16) | 1.01 (0.99-1.02) | 544 | 1.28 (1.08-1.53) | 1.03 (1.01-1.05) |

Models adjusted for age, BMI, race, smoking, AHI, T90, arousal index, sleep efficiency, PLMI, insomnia, hypnotics usage, hypertension, diabetes mellitus, CVD, COPD, chronic kidney disease, liver disease, cancer, and depression.

^a^Models further adjusted for PAP acceptance.

AHI, apnea-hypopnea index; BMI, body mass index; COPD, chronic obstructive pulmonary disease; CVD, cardiovascular disease; ESS, Epworth Sleepiness Scale; OSA, obstructive sleep apnea; PAP, positive airway pressure; PLMI, periodic limb movement index; T90, total sleep time (in percent) spent with SpO_2_ below 90%.

**Supplementary Table 9. Sensitivity analysis comparing characteristics of OSA patients with and without ESS.**

| **Characteristic** | **Total**  **(n = 15,772)** | **With ESS**  **(n = 14,823)** | **Without ESS**  **(n = 949)** | **P value** |
| --- | --- | --- | --- | --- |
| Age, years | 61 (51, 70) | 61 (51, 70) | 62 (53, 72) | 0.001 |
| Men, n (%) | 9,654 (61.2) | 9,081 (61.3) | 573 (60.4) | 0.588 |
| White, n (%) | 14,285 (92.2) | 13,504 (92.7) | 781 (84.9) | < 0.001 |
| BMI, kg/m^2^ | 32.6 (28.7, 37.6) | 32.6 (28.7, 37.6) | 32.2 (28.4, 37.6) | 0.157 |
| Smoking history, n (%) | 4,883 (31.2) | 4,589 (31.2) | 294 (31.2) | 0.995 |
| Sleep measures |  |  |  |  |
| Split-night, n (%) | 14,913 (94.6) | 14,039 (94.7) | 874 (92.1) | 0.001 |
| Total recording time, min | 229 (187, 280) | 229 (187, 281) | 223 (181, 279) | 0.150 |
| Total sleep time, min | 158 (132, 194) | 158 (132, 194) | 154 (129, 186) | 0.002 |
| Sleep efficiency, % | 75.5 (62.4, 85.1) | 75.5 (62.5, 85.1) | 74.9 (60.7, 84.4) | 0.047 |
| Arousal index, events/h | 36.0 (23.6, 55.1) | 35.9 (23.6, 55.0) | 37.4 (24, 57.4) | 0.121 |
| PLMI, events/h | 15.1 (0.0, 52.8) | 15.3 (0.0, 52.8) | 12.6 (0.0, 52.1) | 0.189 |
| AHI, events/h | 17 (9, 35) | 17 (8, 35) | 19 (9, 39) | 0.004 |
| RDI, events/h | 27 (15, 48) | 27 (15, 48) | 29 (16, 51) | 0.060 |
| Mean SpO_2_, % | 93 (91, 94) | 93 (91, 94) | 93 (91, 94) | 0.111 |
| Minimum SpO_2_, % | 83 (78, 86) | 83 (78, 86) | 82 (77, 86) | < 0.001 |
| T90, % | 4.8 (1.2, 17.8) | 4.7 (1.1, 17.5) | 6 (1.3, 22.5) | 0.001 |
| OSA severity |  |  |  | 0.072 |
| AHI 5-14.9, n (%) | 7,013 (44.5) | 6,624 (44.7) | 389 (41.0) |  |
| AHI 15-29.9, n (%) | 3,961 (25.1) | 3,714 (25.1) | 247 (26.0) |  |
| AHI ≥ 30, n (%) | 4,798 (30.4) | 4,485 (30.3) | 313 (33.0) |  |
| Comorbidities |  |  |  |  |
| Hypertension, n (%) | 7,655 (48.5) | 7,157 (48.3) | 498 (52.5) | 0.012 |
| Diabetes mellitus, n (%) | 3,356 (21.3) | 3,083 (20.8) | 273 (28.8) | < 0.001 |
| CVD, n (%) | 4,651 (29.5) | 4,292 (29.0) | 359 (37.8) | < 0.001 |
| COPD, n (%) | 1,123 (7.1) | 1,015 (6.8) | 108 (11.4) | < 0.001 |
| Chronic kidney disease, n (%) | 1,195 (7.6) | 1,071 (7.2) | 124 (13.1) | < 0.001 |
| Liver disease, n (%) | 1,124 (7.1) | 1,042 (7.0) | 82 (8.6) | 0.061 |
| Cancer, n (%) | 1,756 (11.1) | 1,634 (11.0) | 122 (12.9) | 0.082 |
| Depression, n (%) | 523 (3.3) | 495 (3.3) | 28 (3.0) | 0.517 |
| Insomnia, n (%) | 2,185 (13.9) | 2,049 (13.8) | 136 (14.3) | 0.661 |
| Hypnotics usage, n (%) | 1,478 (9.4) | 1,384 (9.3) | 94 (9.9) | 0.560 |
| Follow up, years | 6.1 (4.5, 8.0) | 6.2 (4.5, 8.1) | 5.3 (3.8, 7.2) | < 0.001 |
| PAP acceptance, % | 8,546 (54.5) | 8,003 (54.3) | 543 (57.5) | 0.052 |

Data from continuous variables are reported as median (IQR) values and count (%).

Abbreviations: AHI, apnea-hypopnea index; BMI, body mass index; COPD, chronic obstructive pulmonary disease; CVD, cardiovascular disease; ESS, Epworth Sleepiness Scale; IQR, interquartile range; OSA, obstructive sleep apnea; PAP, positive airway pressure; PLMI, periodic limb movement index; RDI, respiratory disturbance index; SpO_2_, oxyhemoglobin saturation; T90, total sleep time (in percent) spent with SpO_2_ below 90%.

Numbers may not sum to totals because of missing data.

P-values from Mann-Whitney U test or Pearson Chi square test.
